# Comparison of efficacy and safety of traditional Chinese patent medicines for diabetic nephropathy: A protocol for Bayesian network meta-analysis

**DOI:** 10.1097/MD.0000000000029152

**Published:** 2022-05-20

**Authors:** Shilin Liu, Andong Li, Bin Jiang, Jia Mi, Hongmei Nan, Pengjie Bao, Zheng Nan

**Affiliations:** a Changchun University of Chinese Medicine, 1035 Bo Shuo Road, Changchun City, Jilin Province, China; b Endocrinology, First Affiliated Hospital to Changchun University of Chinese Medicine, 1478 Gongnong Road, Changchun City, Jilin Province, China.

**Keywords:** diabetic nephropathy, network meta-analysis, protocol, safety, systematic review, traditional Chinese patent medicine

## Abstract

**Background:**

Diabetic nephropathy (DN) is one of the most serious complications of diabetes. It has become a global public health problem among humans. DN is the leading cause of end-stage renal disease. At present, there is no specific medicine or modern medicinal therapy. In recent years, studies have shown that traditional Chinese patent medicines have been effective in treating DN, with few side effects. There is no systematic review on the treatment of DN with Chinese patent medicines. The current systematic review aims to evaluate the efficacy and safety of Chinese patent medicines for the treatment of DN.

**Methods:**

We will develop a search strategy to search major Chinese and English databases from inception to February 25, 2022 for randomized controlled trials examining the use of traditional Chinese patent medicine for the treatment of DN. The search will be conducted in accordance with the participants, interventions, comparisons, outcomes (PICOS) framework. Two researchers will use EndnoteX9 software to extract data and independently evaluate the quality of the included trials. Finally, the Bayesian network meta-analysis will be carried out by using software such as ReviewManager, Stata16.0, and WinBUGS1.4.3.

**Results:**

The primary outcomes will be urine albumin excretion rate, urea nitrogen, serum creatinine, total effective rate, and adverse events, and the secondary outcomes will be body mass index, fasting blood glucose, and 2-hPG during 75-g OGTT. These outcomes will be examined to provide a reliable basis for the treatment of DN with different traditional Chinese patent medicines.

**Conclusion:**

This review will compare the efficacy and safety of different traditional Chinese patent medicines for treating DN. The results of the study will provide a basis for the selection of adjuvant treatment options for DN.

## 1 Introduction

Diabetic nephropathy (DN), also known as diabetic kidney disease (DKD), is a type of chronic kidney disease caused by diabetes mellitus. The early onset of the disease appears hidden, develops rapidly, and cannot be reversed. DKD is considered one of the leading causes of death in patients with DN, in addition to cardiovascular and cerebrovascular complications.^[1]^ DKD is the main cause of end-stage renal disease (ESRD) in developed and developing countries. Approximately 30% to 50% of ESRD cases worldwide are caused by DKD,^[2]^ which has placed a considerable medical burden on society. DN is thought to be associated with metabolic status, oxidative stress, immune inflammatory factors, genetic factors, and hemodynamic changes. At this stage, modern medicine, apart from general life intervention, is symptomatic treatment. There are no specific drugs or therapies for DKD. When patients approach ESRD, renal replacement therapy is often used. In recent years, traditional Chinese patent medicines have become increasingly accepted and widely used in the treatment of DN. Traditional Chinese patent medicines are a kind of traditional Chinese medicine preparation that has been developed by doctors after many years of clinical research and has achieved certain curative effects as an auxiliary medicine combined with conventional Western medicine therapy to treat DN. These medicines include Huangkui capsules^[3]^, bailing capsules, Niaoduqing granules,^[4]^ naoxintong capsules,^[5]^ qishao capsules,^[6]^ Tangweikang capsules,^[7]^ Jinshuibao capsules,^[8]^ Shenqi Jiangtang granules,^[9]^ Sanhuang Yishen capsules,^[10]^ and so on.^[3]^ The treatment guidelines^[11]^ also emphasize the important role of Huangkui capsules and Keluoxin capsules in the treatment of the disease. However, there is no direct comparison of the efficacy and safety of traditional Chinese patent medicines in treating DN. There is also a lack of objective and rigorous comparisons between clinical trials of traditional Chinese patent medicines. Therefore, it is very difficult to carry out rigorous statistical analyses and appraisals of the curative effect of each traditional Chinese patent medicine, which also inhibits clinicians from making evidence-based decisions regarding the best medication. Network meta-analysis (NMA), as an extension of traditional meta-analysis, is able to analyze the relative effectiveness of different interventions through an indirect comparison of common reference groups.^[12]^ The Bayesian method is the mainstream statistical model of reticular meta-analysis, with more accurate estimation and flexible modeling. A combination of direct and indirect evidence from the NMA can be used to enhance the relative validity of the inferential estimates and across multiple ranked interventions.^[13]^ Therefore, this study used Bayesian modeling based on NMA to compare the clinical efficacy of different oral traditional Chinese patent medicines among patients with DN. This method can not only provide direct clinical evidence for the treatment of DN with traditional Chinese patent medicine but also provide a better clinical research method for the further selection of effective interventions for DN .

## 2 Materials and methods

### 2.1 Study registration

This study is a retrospective study and NMA. Our goal is to publish this review in a peer-reviewed journal. Therefore, there is no need for patient and public participation, informed consent, or ethical approval in the design, process, and outcome of the study. The study will be conducted in accordance with the PRISMA-P guidelines.^[14]^ The agreement has been approved by the Open Scientific Framework Registry (https://osf.io/7m4bk). The registration number is: INPLASY202220114 (DOI 10.37766/inplasy2022.2.0114).

### 2.2 Inclusion criteria

#### 2.2.1 Type of research

The inclusion criteria are as follows: studies focused on traditional Chinese patent medicine treatment of DN; randomized controlled trials; and studies published in Chinese or English. There are no restrictions regarding publication date or blinding methods.

#### 2.2.2 Types of patients

Patients who meet the diagnostic criteria of DN, including the criteria issued by the American Diabetes Association^[15]^ in 2009 and the Mogensen staging^[16]^ of DN. There will be no restrictions on sex, race, age, course of disease, or Traditional Chinese Medicine syndrome.

#### 2.2.3 Interventions

The control group will include patients treated with Western medicine. The experimental group will include patients treated with traditional Chinese patent medicines. The use of traditional Chinese patent medicines is limited to oral administration. There will be no restrictions regarding the time, frequency, and dosage of medication.

#### 2.2.4 Outcomes

1. Primary outcomes: Urinary albumin excretion rate, urea nitrogen, serum creatinine, total effective rate, and adverse events (e.g., hypoglycemia, gastrointestinal symptoms, rash).
2. Secondary outcomes: body mass index, fasting blood glucose, 2-hPG during 75-g OGTT, HbA1c, 24-hour urine protein quantification, fasting insulin, and 2-hour postprandial insulin.

### 2.3 Exclusion criteria

The exclusion criteria are as follows:

1. The patients who do not have DN; type 2 diabetes with diabetic ketoacidosis; urinary tract infections; severe heart, lung, or liver disease; or on dialysis.
2. The experimental group was given treatments other than traditional Chinese patent medicines, or the control group was not treated with Western medicine.
3. The type of study is not a randomized controlled trial (e.g., animal studies, conference papers, reviews, case studies, and duplicate studies).
4. Literature without major outcome indicators or incomplete data that could not be obtained.

### 2.4 Database and search strategy

We have undergone specialized training on literature search methods. After 2 pre-checks, the retrieval strategy will be formulated. The PubMed, Embase, Cochrane Library, Web of Science, Chinese Biomedical Literature Database (CBM), Chinese National Knowledge Infrastructure (CNKI), Chinese Scientific Journal Database (VIP), and Wanfang databases will be searched up to February 25, 2022. We will use a combination of subject words and free words for each database. In addition, we will examine ongoing and unpublished studies registered with the World Health Organization's International Clinical Trial Registry. Moreover, we will manually search the reference lists of all relevant systematic reviews to identify additional eligible studies. The specific retrieval strategy for PubMed is shown in Table 1. The data will also be retrieved from other sources.

**Table 1:** Detailed search strategy for Pubmed.

Number	Search item
#1	Diabetic nephropathy[MeSH]
#2	Nephropathies, Diabetic[Title/Abstract] OR Nephropathy, Diabetic[Title/Abstract] OR Diabetic Nephropathy[Title/Abstract] OR Diabetic Kidney Disease[Title/Abstract] OR Diabetic Kidney Diseases[Title/Abstract] OR Kidney Disease, Diabetic[Title/Abstract] OR Kidney Diseases, Diabetic[Title/Abstract] OR Diabetic Glomerulosclerosis[Title/Abstract] OR Glomerulosclerosis, Diabetic[Title/Abstract] OR Intracapillary Glomerulosclerosis[Title/Abstract] OR Nodular Glomerulosclerosis[Title/Abstract] OR Glomerulosclerosis, Nodular[Title/Abstract] OR Kimmelstiel-Wilson Syndrome[Title/Abstract] OR Kimmelstiel Wilson Syndrome[Title/Abstract] OR Syndrome, Kimmelstiel-Wilson[Title/Abstract] OR Kimmelstiel-Wilson Disease[Title/Abstract] OR Kimmelstiel Wilson Disease[Title/Abstract]
#3	#1 OR #2
#4	Complementary Therapies[MeSH]
#5	Therapies, Complementary[Title/Abstract] OR Therapy, Complementary[Title/Abstract] OR Complementary Medicine[Title/Abstract] OR Medicine, Complementary[Title/Abstract] OR Alternative Medicine[Title/Abstract] OR Medicine, Alternative[Title/Abstract] OR Alternative Therapies[Title/Abstract] OR Therapies, Alternative[Title/Abstract] OR Therapy, Alternative[Title/Abstract]
#6	Capsule[Title/Abstract] OR Pill[Title/Abstract] OR Powder[Title/Abstract] OR Pulvis[Title/Abstract] OR Tablet[Title/Abstract] OR Particle[Title/Abstract]
#7	Huangkui capsule[Title/Abstract] OR Keluoxin capsule[Title/Abstract] OR Yuquan pill[Title/Abstract] OR Bailing capsule[Title/Abstract] OR Jinshuibao capsule[Title/Abstract] OR Jinshuibao tablet[Title/Abstract] OR Niaoduqing granule[Title/Abstract] OR Shenshuaining capsule[Title/Abstract] OR Kunxian capsule[Title/Abstract] ORTripterygium wilfordii polyglycosides tablets[Title/Abstract] OR Tangmaikang granule[Title/Abstract] OR Nephritis rehabilitation tablet[Title/Abstract] OR Qishao capsule[Title/Abstract] OR Zhaohua Xiaoshen’an capsule[Title/Abstract] OR Tangsheningtang[Title/Abstract] OR Weikang capsule[Title/Abstract] OR Xihongkang[Title/Abstract] OR Tangshenkang capsule[Title/Abstract] OR Tangjiangshenkang granule[Title/Abstract] OR compound Danshen dropping Pill
#8	#4 OR #5 OR #6 OR #7
#9	Randomized controlled trial[Publication Type] OR Controlled clinical trial[Publication Type]
#10	Randomized[Title/Abstract] OR randomly[Title/Abstract]
#11	#9 OR #10
#12	#3 AND #8 AND #11

### 2.5 Data extraction

**Figure 1:**
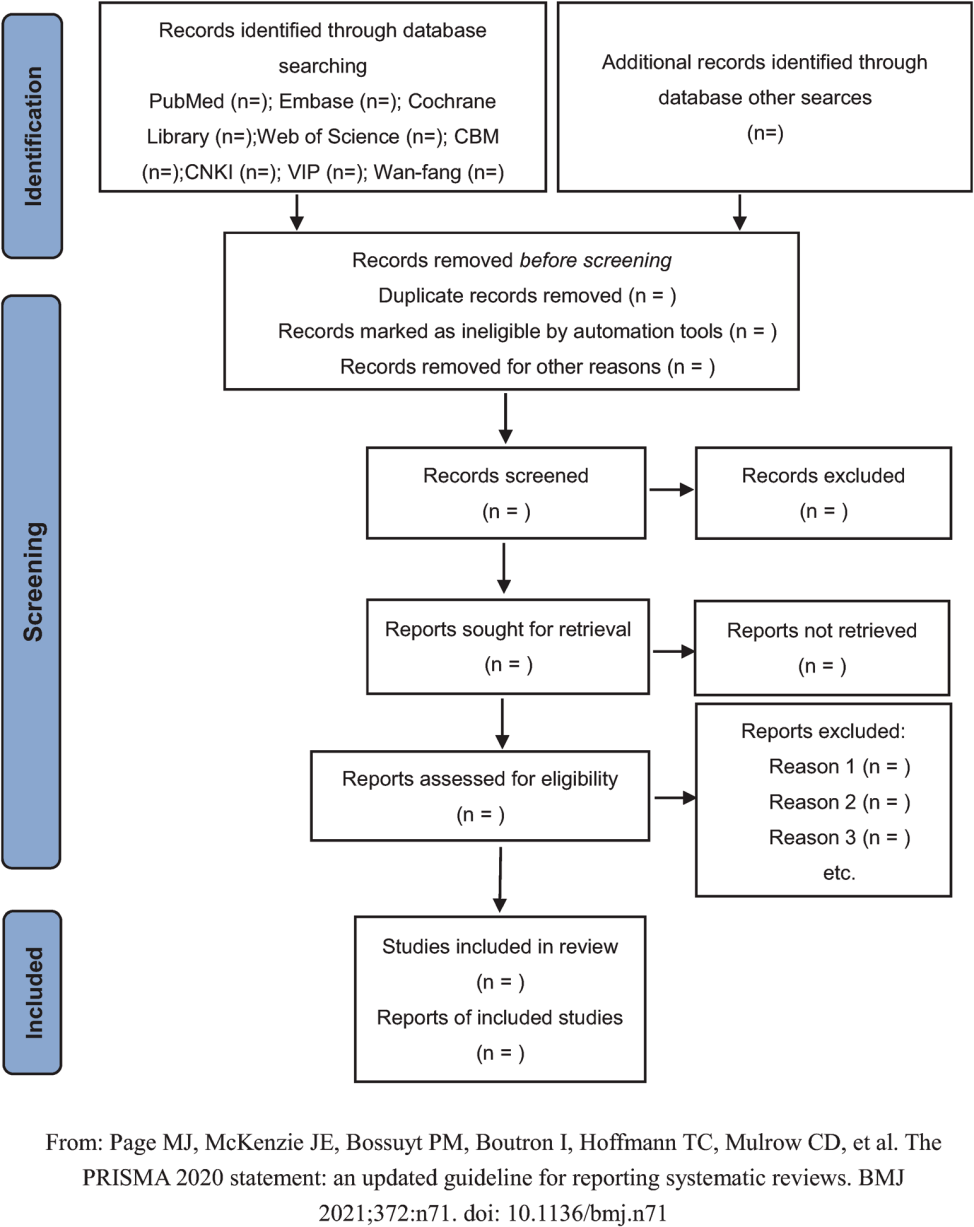
Flow diagram of study selection process. From: Page MJ, McKenzie JE, Bossuyt PM, Boutron I, Hoffmann TC, Mulrow CD, et al. The PRISMA 2020 statement: an updated guideline for reporting systematic reviews. BMJ 2021;372:n71. doi: 10.1136/bmj.n71.

Two medical researchers (Shilin Liu and Andong Li) will search the literature based on a predetermined search strategy. Endnote X9 software (Clarivate) will be used to extract the following data according to the inclusion and exclusion criteria: the basic information of the literature, the basic characteristics of patients, intervention measures, outcome indicators, and so on. We will exclude all meeting records, newspapers, guides, letters, and other literature. Disagreements between the 2 reviewers will be resolved by consulting a third researcher (Zheng Nan). When the full text or analysis of the literature reveals incomplete or missing information that may affect the results of this analysis, we will attempt to contact the authors of the literature for data. The PRISMA flow chart^[17]^ for this study is shown in Figure 1.

### 2.6 Risk of bias assessment

The 2 researchers will independently evaluate the methodological quality of the included studies using the Cochrane risk-bias assessment tool. The following criteria will be evaluated: random sequence generation method; whether allocation concealment was used; whether the subject and the intervention provider were blinded; whether the result evaluator was blinded; whether the result data were complete; whether selective results were reported; and other sources of bias. According to the relevant assessment criteria, the included studies will be rated as low risk of bias, high risk of bias, or uncertain of bias risk. If there are disagreements regarding the quality assessments, a third researcher will be consulted.

### 2.7 Assessment of heterogeneity

Because of the diversity of our research design, the included literature will be from different regions or countries, and it is inevitable that there will be differences. In this study, we will use I^2^ and X^2^ statistics to evaluate the statistical heterogeneity between studies. If I^2^ is between 50% and 100%, there is statistical heterogeneity, and we will use a random effects model to analyze the data. If I^2^ ≤ 50%, the heterogeneity test is not significant, and the fixed effects model will be used. In addition, if there is differences, we will conduct subgroup or sensitivity analyses to identify the source of the heterogeneity.

#### 2.7.1 Subgroup analysis

Subgroup analysis will be used to explore the sources of heterogeneity, including analyses based on race, age, country, sex, dosage form, and different forms of intervention.

#### 2.7.2 Sensitivity analysis

Sensitivity analysis will be carried out by excluding each study one at a time. If the heterogeneity changes when a study is removed, it may be considered a source of heterogeneity. We will further analyze and explain why this study is a source of heterogeneity. If the heterogeneity remains unchanged after excluding studies, the results of our study will be considered relatively robust.

### 2.8 Statistical analysis

#### 2.8.1 Statistical model selection

We will use Review Manager software (REVMAN v5.3 Cochrane Collaboration) and STATA 16.0 software (StataCorp LLC) to perform a meta-analysis of the included literature. *P* < .05 will be considered statistically significant. The 2 researchers will enter and account for the data independently, and a third researcher will review the data. The pooled effects will be represented by the standardized mean square deviation and 95% confidence interval (CI). The combination effect was expressed by the odds ratio and 95% CI.

#### 2.8.2 Network meta-analysis

The NMA will be conducted using STATA 16.0 software, and the random effects model will be used to merge data and create a network graph to show the direct and indirect comparisons between different interventions. In the network, the larger the arm is, the larger the basic data, and the larger the circle area is, the stronger the effect of the intervention. Bayesian NMA is based on the Markov-chain-Monte-Carlo (MCMC) method. We will use the MCMC method in WinBUGS1.4.3 (Biostatistics the Medical Research Council, Cambridge, UK)^[18]^ to analyze the random effects model with Bayesian mesh meta. We will use 3 MCMCs to simulate. The number of iterations will be set to 100,000, and we will use the first 5000 iterations for annealing to eliminate the effect of the initial value. The consistency of each closed loop will be evaluated by calculating the relative ratio and its 95% CI. A lower limit of 95% CI equal to 1 indicates that the consistency is high. If the relative ratio is close to 1, the direct evidence is consistent with the indirect evidence, and the fixed effect model should be used for analysis. Otherwise, it is considered that there is obvious inconsistency in the closed loop, and the random effect model should be used for analysis.^[19]^ The data of the 2 categories will be expressed as odds ratios and 95% CIs, and statistical significance of the differences will be examined.^[20]^ The curative effect of different intervention measures will be ranked by WinBUGS 1.4.3 software, and the area under the curve will be recorded. The area under the curve is expressed as a percentage, and the larger the percentage is, the better the therapeutic effect.

### 2.9 Assessment of inconsistency

When there is a closed loop in the mesh meta-analysis, its consistency needs to be evaluated. Therefore, we will use the node splitting method to calculate the differences between direct comparison evidence and indirectly compare the evidence to determine whether there is inconsistency.

### 2.10 Publication bias and evidence quality assessment

When analyzing the effect index, if the number of articles included is more than 10, then a funnel chart can be used to analyze the publication bias risk. When the funnel diagram is obviously asymmetric, it indicates that there is publication bias. The reliability of the evidence will be assessed through the grading recommendations for evaluation, development, and evaluation framework. The quality of the evidence will be classified as high, medium, low, or very low.

## 3 Discussion

With the continuous improvement of people's living standards, the incidence of DN is increasing year by year. DN is the main cause of end-stage kidney disease. Modern medical treatment is limited in many ways. The prognosis of DN is poor, and aggravation of the disease can shorten the natural lifespan of patients. Approximately 25% of patients can develop end-stage renal failure within 6 years, 50% within 10 years, and 75% within 15 years. The average time from the onset of proteinuria to death from uremia is approximately 10 years. A number of studies have shown that traditional Chinese patent medicines can effectively assist in the treatment of DN, relieve symptoms of patients with DN, reduce proteinuria, and improve renal function. Huangkui capsules^[21]^ can reduce EMT in renal tubules of DN model rats. Shenshuaining capsules^[22]^ can significantly reduce serum urea nitrogen and creatinine and improve renal function. Gushen Jiedu capsules^[23]^ can inhibit apoptosis and protect the kidney in rats with DN. Yishen capsules^[24]^ promote podocyte autophagy and improve DN by regulating the SIRT1/NF-kappaB signaling pathway. Jinlida granules^[25]^ can not only alleviate the clinical symptoms of patients with DN but also downregulate the expression of VEGF and IGF-1 in serum. Although there are clinical reports on the efficacy of traditional Chinese patent medicines in the treatment of DN,^[26]^ they are not comprehensive and systematic. To the best of our knowledge, this study is the first to use Bayesian mesh meta-analysis on the basis of existing research and to evaluate and rank the advantages of various traditional Chinese patent medicines in the treatment of DN. The results of this study will provide a basis for clinical rational drug use, clinical planning, and medical insurance catalog screening. Notably, the quality of our reticular meta-analysis may be limited by the quality of basic data and may be affected by publication bias. Therefore, it is still necessary to conduct high-quality, multicenter research in the future to verify the effectiveness and safety of traditional Chinese patent medicines in the treatment of DN.

## Author contributions

**Conceptualization:** Shilin Liu, Zheng Nan.

**Data curation:** Shilin Liu, Andong Li.

**Funding acquisition:** Jia Mi, Hongmei Nan, Zheng Nan.

**Methodology:** Andong Li, Bin Jiang.

**Project administration:** Jia Mi, Hongmei Nan, Zheng Nan.

**Software:** Pengjie Bao.

**Writing – original draft:** Shilin Liu.

**Writing – review & editing:** Shilin Liu, Andong Li.
